# Population Structure and Distribution Patterns of the Sibling Mosquito Species *Culex pipiens* and *Culex torrentium* (Diptera: Culicidae) Reveal Different Evolutionary Paths

**DOI:** 10.1371/journal.pone.0102158

**Published:** 2014-07-21

**Authors:** Antje Werblow, Sven Klimpel, Sarah Bolius, Adriaan W. C. Dorresteijn, Jan Sauer, Christian Melaun

**Affiliations:** 1 Goethe-University (GU), Institute for Ecology, Evolution and Diversity; Biodiversity and Climate Research Centre (BiK-F), Senckenberg Gesellschaft für Naturforschung (SGN), Frankfurt am Main, Germany; 2 Institute for General Zoology and Developmental Biology, Justus Liebig University (JLU), Giessen, Germany; 3 Department of Chemical Ecology, Bielefeld University, Bielefeld, Germany; Institut Pasteur, France

## Abstract

Nowadays a number of endemic mosquito species are known to possess vector abilities for various diseases, as e.g. the sibling species *Culex pipiens* and *Culex torrentium*. Due to their morphological similarity, ecology, distribution and vector abilities, knowledge about these species' population structure is essential. Culicidae from 25 different sampling sites were collected from March till October 2012. All analyses were performed with aligned cox1 sequences with a total length of 658 bp. Population structure as well as distribution patterns of both species were analysed using molecular methods and different statistical tests like distance based redundancy analysis (dbDRA), analysis of molecular variances (AMOVA) or McDonald & Kreitman test and Tajima's D. Within both species, we could show a genetic variability among the cox1 fragment. The construction of haplotype networks revealed one dominating haplotype for *Cx. pipiens*, widely distributed within Germany and a more homogeneous pattern for *Cx. torrentium*. The low genetic differences within *Cx. pipiens* could be a result of an infection with *Wolbachia* which can induce a sweep through populations by passively taking the also maternally inherited mtDNA through the population, thereby reducing the mitochondrial diversity as an outcome of reproductive incompatibility. Pairwise population genetic differentiation (F_ST_) ranged significantly from moderate to very great between populations of *Cx. pipiens* and *Cx. torrentium*. Analyses of molecular variances revealed for both species that the main genetic variability exists within the populations (*Cx. pipiens* [88.38%]; *Cx. torrentium* [66.54%]). Based on a distance based redundancy analysis geographical origin explained a small but significant part of the species' genetic variation. Overall, the results confirm that *Cx. pipiens* and *Cx. torrentium* underlie different factors regarding their mitochondrial differentiation, which could be a result of endosymbiosis, dispersal between nearly located populations or human introduction.

## Introduction

Since the late 19th century, mosquitoes are known as vectors for various diseases as malaria, dengue, yellow or Chikungunya fever [1], [2]. However the neglect of research on mosquitoes has resulted in little knowledge about mosquito fauna and its vector competence, especially in Germany. Only during recent years, research in this field has been continued and intensified. Many mosquito species are extremely adaptable to changing climate conditions or the consequences of urbanization [3], which has already led to the expansion of species' distribution, at least for some species. In addition, the spread is encouraged by the increasing international travel and global freight transportation which have direct influence on the introduction and establishment of mosquito-associated viruses from other countries to Europe [4]–[6]. Many studies deal with invasive species such as *Aedes aegypti*, *Aedes albopictus* or *Ochlerotatus japonicus*, which have been introduced and established in different countries. However, numerous indigenous mosquito species are known to be potential carriers of diseases such as Sindbis virus, Ockelbo virus, Usutu virus, Batai virus, West-Nile virus or even malaria [2], [7]–[11].

In this context the genus *Culex* with more than 750 described species worldwide [3] is of high medical and veterinary interest. Its members are vectors for various diseases, and occur in the proximity of human dwellings [7]. Within the genus *Culex*, the subgenus *Culex* includes seven species in Europe [12], with *Cx. pipiens* being one of the most common and widespread holarctic species. Together with its palaearctic biotypes *Cx. pipiens pipiens* and *Cx. pipiens molestus*, *Cx. pipiens* belongs to the *Culex pipiens* complex which also includes the non-european species *Cx. quinquefasciatus*, *Cx. australicus* as well as *Cx. globocoxitus* [13]. Females of *Cx. pipiens pipiens* are known to be ornithophilic, but several studies also mention a potential anthropophilic diet (e.g. [14]). Furthermore they are anautogenous, eurygamous and diapausing during wintertime. After diapause, females lay egg batches of 150–240 eggs on the water surface where the larvae hatch within one or two days. Depending on climate conditions larval development takes one week up to several weeks with several generations per year [3]. The larvae of *Cx. pipiens* can be found in nearly every natural, artificial, permanent or semi-permanent water body as well as in rural or urban areas [15], [16].

*Culex torrentium* another common species is considered to be the sister-taxon of *Cx. pipiens* [17]. The differentiation of larvae and females of both species is extremely difficult resulting in wrong determination or neglect [18], [19]. They share comparable ecological characteristics regarding the habitat of the adults as well as breeding sites and an almost identical morphology [18]. The only reliable distinguishable morphological characteristic is the structure of the male hypopygium [16]. Thus, it is not always certain that *Cx. torrentium* and *Cx. pipiens* were correctly differentiated in publications of past decades, where they were also often only collectively evaluated as bundles of “*Cx. pipiens*/*torrentium*” [20], [21]. As a consequence, European abundance and distribution of *Cx. pipiens* and *Cx. torrentium* was solely based on few identified males, and the distribution of both species is largely unknown [16], [22] with most existing data being limited to Scandinavia and Russia [14], [17], [21], [22]. A detailed knowledge of the distribution of both species is essential as both are able to transmit a variety of diseases [13]. Notably *Cx. pipiens* is a vector for the West Nile virus which has become the most important mosquito-borne virus during the last 20 years in the warmer regions of Europe [2]. Usually, the virus is transmitted in an avian cycle, but it is also responsible for an increasing number of human infections [2], [23]. The symptoms vary from fever to coma and paralysis [24], [25]. *Culex pipiens* is also a vector of different encephalitis diseases and Rift valley fever [17]. Experimental studies detected *Cx. pipiens* and *Cx. torrentium* as potential vectors of Sindbis and Ockelbo virus, with *Cx. torrentium* showing a significantly higher vector competence in the laboratory and seeming to be the main enzootic vector for Sindbis virus in Sweden [12], [22]. Because of the medical importance and the unsatisfactory morphological differentiation of both species, clear identification methods are of great interest. To enable an unequivocal classification several PCR-based assays have been developed using different molecular genetic markers e.g., ace-2 or ITS2 [13], [17], [26]. Vinogradova and Shaikevich [27] make use of the *Wolbachia* infection in *Cx. pipiens* (inherited maternally) in order to distinguish this type from *Cx. torrentium*. Recently a multiplex real-time PCR for simultaneous detection and differentiation of *Cx. pipiens* biotypes and *Cx. torrentium* was established [28]. Data retrieved from DNA sequences are largely used in molecular taxonomy e.g. for defining the genetic structure of vector species populations, for resolving phylogenetic relationships among and within groups of Culicidae [29]–[32], but also for the identification of species [33]–[35]. For molecular species identification, a fragment of the cytochrome c oxidase subunit I (cox1) mitochondrial gene has been used commonly for taxon barcoding and for assessing genetic divergence among closely related species [36], [37]. This fragment was also used to analyse species complexes as well as to compare phylogeographic patterns within closely related species (e.g. [38], [39]). One problem regarding the cox1 DNA barcode is the ambiguous identification or the absence of clusters in trees of recently diverged species [40], [41]. Therefore new algorithms have been developed for improvement of these subjects (e.g. [42], [43]).

A former study about *Cx. pipiens* and *Cx. torrentium* within the Frankfurt/Rhine-Main Metropolitan Region showed a genetic variability within both species [44]. Based on these results, a nationwide survey was started to analyse the distribution of both species, as well as their sympatric occurrence. The aim of the current study was to analyse the population structure of *Cx. pipiens* and *Cx. torrentium* and to investigate whether there are differences in their genetic composition and patterns of distribution. Here, we present first-time population comparisons for both species in Germany.

## Methods

### Sampling

**Figure 1:**
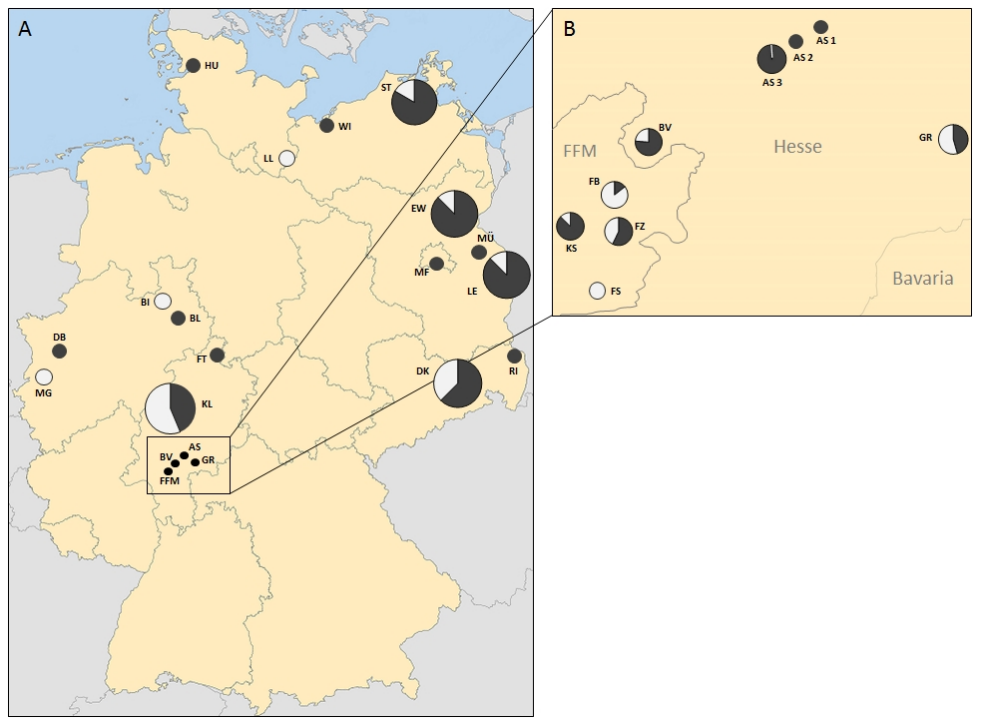
Distribution of *Culex torrentium* (white) and *Culex pipiens* (grey) in Germany (A) and the Hessian Rhine-Main area (B). Small circles in Figure 1A (excluding the circles for FFM, BV, AS and GR) indicate that only one of the two species was detected at this specific locality. Pie charts indicate the ratio of the two detected species at this locality. The sizes of the pie chart and the circles do not relate to the number of investigated individuals (see Table 1). A: Overview of the sampling localities across Germany. Abbreviations: AS = Altenstadt, BV = Bad Vilbel, MF = Berlin-Marienfelde, BI = Bielefeld, BL = Bad Lippspringe, DB = Duisburg, DK = Dresden-Klotzsche, EW = Eberswalde, FFM = Frankfurt/Main (four different localities: Bornheim (FB), Bockenheim (KS), Sachsenhausen (FS) and Ostend (FZ)), FT = Fuldatal, GR = Gründau-Rothenbergen, HU = Husum, KL = Klein Linden, LE = Lebus, LL = Langenlehsten, MG = Mönchengladbach, MÜ = Müncheberg, RI = Rietschen, ST = Stralsund and WI = Wismar. B: Detailed view of the Rhine-Main area with Höchst a.d.N. (A1), Eichen (AS2), Heldenbergen (AS3), Klein Linden. Map was created with ArcMap 10.1.

Culicidae from 25 different sampling sites in 22 German cities (see Table 1 and Figure 1) were collected from March until October 2012. Collection sites were in rural as well as in urban areas near human dwellings (specific information about the sampling sites are in Table 1). Adults were collected using BG-Sentinel traps (Biogents AG, Regensburg, Germany) with CO_2_ and/or BG Lure as an attractant as well as EVS-traps with dry ice. Caught specimens were stored at −20°C. Larvae were collected from natural as well as artificial water pools using hand nets or ovitraps, fixed and preserved in 70% ethanol or kept alive to raise them to adults. For morphological identification (based on [3], [45], [46]) of larvae and adults (to genus level), a stereomicroscope was used. Names and addresses of persons who conducted the trapping as well as permission numbers (where permission was necessary) can be provided on request (see also Table S1). No endangered or protected species were involved in this study.

**Table 1:** Coordinates and abbreviations of the 25 analysed sampling localities.

			Coordinates (decimal degrees)					Coordinates (decimal degrees)	
Locality		habitat	latitude	longitude	Locality		habitat	latitude	longitude
**Bad Lippspringe**	**BL**	house and garden	51.790131	8.829283	**Gründau-Rothenbergen**	**GR**	forest near to the stream Kinzig	50.185181	9.092017
**Bad Vilbel**	**BV**	water butt	50.182336	8.739481	**Heldenbergen**	**AS 3**	trees near to open grasland	50.245517	8.883197
**Berlin-Marienfelde**	**MF**	open space near to trees and ponds	52.398391	13.3658	**Höchst a.d. Nidder**	**AS 1**	trees near to open grasland	50.266197	8.935378
**Bielefeld**	**BI**	Open space near to ponds	52.043738	8.479485	**Husum**	**HU**	golf court	54.487004	9.095349
**Dresden**	**DK**	Forest, heathland	51.128926	13.792856	**Klein Linden**	**KL**	backyard	50.556379	8.637328
**Duisburg**	**DB**	backyard	51.472747	6.773101	**Langenlehsten**	**LL**	backyard	53.5	10.733333
**Eberswalde**	**EW**	backyard	53.893335	11.45868	**Lebus**	**LE**	trees near to parking spot	52.403069	14.529469
**Eichen**	**AS 2**	trees near to the stream Nidder	50.254558	8.906103	**Mönchengladbach**	**MG**	water butt	51.148595	6.450997
**Frankfurt-Bockenheim**	**KS**	tub with hay water in backyard	50.116232	8.64006	**Müncheberg**	**MÜ**	forest, wetland	52.516691	14.104214
**Frankfurt-Bornheim**	**FB**	cemetery with coniferous trees	50.138216	8.704802	**Rietschen**	**RI**	backyard	51.396427	14.786003
**Frankfurt-Ostend**	**FZ**	within trees near to a pond	50.116254	8.701958	**Stralsund**	**ST**	water butt backyard	54.325172	13.081279
**Frankfurt-Sachsenhausen**	**FS**	city forest	50.072015	8.680401	**Wismar**	**WI**	backyard	53.893335	11.45868
**Fuldatal**	**FT**	backyard	51.383333	9.55					

### Molecular species identification

The DNA extraction was carried out with glass fiber plates (Pall GmbH, Dreieich) following a former described protocol [47]. The cytochrome c oxidase subunit 1 gene fragment (cox1) was amplified using the standard barcoding primers LCO 1490 (5′ GGTCAACAAATCATAAAGATATTGG 3′) and HCO 2198 (5′ TAAACTTCAGGGTGACCAAAAAATCA 3′) [48]. PCR reaction mixture contained 10 pmol of each primer, 0.2 nM of each dNTP, PCR buffer, BSA, MgCl_2_, 1U *Taq* polymerase (TrueStart Hot Start, Fermentas) as well as varying concentrations of DNA and Millipore water, in a total volume of 30 µl. The cycle parameters were the following: 1 cycle of initial denaturation at 94°C, 60 s; 6 cycles of 94°C, 40 s; 45°C, 40 s; 72°C, 60 s; 36 cycles of 94°C, 40 s; 51°C, 40 s; 72°C, 60 s and for terminal extension 1 cycle 72°C, 5 min; with a final ramping to 8°C. The yield and quality of DNA was analysed with SYBR-Green (Life Technologies GmbH) staining and agarose gel-electrophoresis. Sequencing and sequence analysis were carried out as previously described [44]. The obtained sequences were deposited in GenBank under accession numbers HF562483-HF562835 and HG793395-HG793655.

### Phylogenetic and Phylogeographic analyses

To infer the population structure of *Cx. pipiens* and *Cx. torrentium* and to analyse the processes that might have shaped the present day distribution, we used the cox1 barcoding fragment, which can distinguish between both species. The McDonald & Kreitman Test was calculated to show neutral evolution or selection among the analysed mitochondrial DNA. In addition we used Tajima's D to test recent demographic or range expansion. We also calculated pairwise F_ST_'s to show differences between haplotype compositions of sampling points and analysed the molecular variances as well as genetic distances and genetic variability depending on geographical origin. All analyses were performed with aligned cox1 sequences with a length of 658 bp where no frame shifts or stop codons were found. The genealogical relationship between haplotypes of *Cx. pipiens* and *Cx. torrentium* was analysed by reconstructing phylogenetic networks for each species. We used the method of statistical parsimony as described by Templeton et al. [49], implemented in the software TCS 1.21 [50].

For population analyses, models of sequence evolution for the population genetic analysis were chosen according to MODELTEST [51] as implemented in MEGA5 [52] and based on Akaike information criterion (AIC). Based on the AIC, the Tamura-3-parameter Model [53] as the best fitting model was used for all analysis. Furthermore, we used this model to calculate genetic distances within and between both species using MEGA5. To further analyse whether the genetic distance, increased with higher geographic distance we used the distance based redundancy analysis (dbDRA) [54], as implemented in DISTLM (distance based multivariate analysis for a linear model) [55], [56]. Using this analysis we could test for signs of isolation by distance (IBD, [57]) in the dataset as implemented in DISTLM. Analyses of molecular variances (AMOVA, [58]) were carried out using Arlequin 3.5.1.2 [59] based on the distance method of Tamura and Nei [60], where data were analysed in a hierarchical manner to estimate variance components at the different spatial scales. The level of genetic differentiation was measured by F_CT_, F_SC_, and F_ST_, which refer to distance among groups, among populations within groups and within populations (group specification see Table 2). For calculations of pairwise F_ST_'s as well as AMOVA analyses, we omitted all populations with less than 5 individuals and grouped the sampling points in Frankfurt to Frankfurt-all (FFM). We tested whether the cox1 sequences evolved neutrally with the McDonald & Kreitman [61] test as implemented in DnaSP version 5.10.01 [62]. We used individuals of *Cx. modestus* as outgroup taxa. We also analysed Tajima's D [63] and calculated population pairwise F_ST_'s, to determine significance by permuting genotypes among populations (1023 permutations), using Arlequin 3.5.1.2. Additionally we tested whether there was a recent range expansion, a bottleneck or a selective sweep within the two species and their populations [57], [64]–[65]. The significances were generated using the implemented permutation test in Arlequin 3.5.1.2.

**Table 2:** AMOVA group structure of *Culex pipiens* and *Culex torrentium*.

*Culex pipiens*		*Culex torrentium*	
Group No.	Locality	Group No.	Locality
**Group 1**	Eichen	**Group 1**	Bad Vilbel
	Höchst		Klein Linden
	Bad Lippspringe		Frankfurt
	Bad Vilbel	**Group 2**	Dresden
	Gründau		Stralsund
	Klein Linden		Langenlehsten
	Frankfurt	**Group 3**	Mönchengladbach
	Heldenbergen	**Group 4**	Gründau
**Group 2**	Dresden		
	Eberswalde		
	Lebus		
	Berlin-Marienflede		
	Rietschen		
	Stralsund		
	Wismar		

Group structures are based on pairwise F_ST_'s of *Culex pipiens* and *Culex torrentium*.

## Results

### Sequence analyses

In total, 597 individuals of *Cx. pipiens* (399 = 250 adults, 120 larvae and 29 pupae) and *Cx. torrentium* (198 = 88 adults, 83 larvae and 27 pupae) from 25 different localities within Germany were sequenced and compared with sequences deposited in the GenBank using the BLAST algorithm [66]. In total *Cx. pipiens* was much more abundant than *Cx. torrentium* and could be detected at 21 out of 25 sampling sites (Figure 1). At ten sites it was the only occurring *Culex* species whereas at 11 sampling sites it co-occurred with *Cx. torrentium*. In contrast *Cx. torrentium* was only detected at 15 out of 25 sampling sites; at four sites it was the only *Culex* species found. In order to identify mutations in the cox1 gene fragment within and between species, the most frequent sequence of each species (*Cx. torrentium* 42.4%, *Cx. pipiens* 90.5%) was used as the reference (H1) for other haplotypes. For *Cx. torrentium* only one to three different haplotypes were observed in 12 out of 15 localities (Table 3) whereas in Klein Linden (n = 11), Langenlehsten (n = 9) and Dresden (n = 8) a higher haplotype diversity was detected. For *Cx. pipiens* the highest haplotype diversity was observed in Heldenbergen and Dresden (both n = 6). A haplotype diversity with more than three different haplotypes at a sampling site was identified in Berlin, Lebus, Rietschen and Stralsund.

**Table 3:** Sampling localities in Germany with abbreviations and number of sequences and detected haplotypes at each locality.

		*Culex pipiens*		*Culex torrentium*				*Culex pipiens*		*Culex torrentium*	
Locality		sequences	haplotypes	sequences	haplotypes	Locality		sequences	haplotypes	sequences	haplotypes
**Bad Lippspringe**	**BL**	5	1 (1)	-	-	**Gründau-Rothenbergen**	**GR**	5	1 (1)	6	3 (11;12;13)
**Bad Vilbel**	**BV**	45	2 (1;7)	14	2 (1;3)	**Heldenbergen**	**AS 3**	66	6 (1;2;3;4;5;6)	1	1 (4)
**Berlin-Marienfelde**	**MF**	17	4 (1;8;19;20)	-	-	**Höchst a.d. Nidder**	**AS 1**	5	1 (1)	-	-
**Bielefeld**	**BI**	-	-	2	1 (2)	**Husum**	**HU**	1	1 (1)	-	-
**Dresden**	**DK**	35	6 (1;2;9;10;11;12)	21	8 (1;2;3;5;6;7;8;9)	**Klein Linden**	**KL**	45	2 (1;2)	58	11 (2;3;8;11;19;20;21;22;23;24;25)
**Duisburg**	**DB**	3	2 (1;8)	-	-	**Langenlehsten**	**LL**	-	-	18	9 (2;3;7;8;14;15;16;17;18)
**Eberswalde**	**EW**	7	2 (1;13)	1	1 (7)	**Lebus**	**LE**	7	4 (1;16;17;18)	1	1 (3)
**Eichen**	**AS 2**	39	1 (1)	-	-	**Mönchengladbach**	**MG**	-	-	50	3 (5;26;27)
**Frankfurt-Bockenheim**	**KS**	55	3 (1;14;15)	8	1 (3)	**Müncheberg**	**MÜ**	4	1 (1)	-	-
**Frankfurt-Bornheim**	**FB**	1	1 (1)	6	2 (3;5)	**Rietschen**	**RI**	22	4 (1;2;21;22)	-	-
**Frankfurt-Ostend**	**FZ**	4	1 (1)	3	3 (1;5;10)	**Stralsund**	**ST**	25	4 (1;21;23;24)	5	3 (1;3;7)
**Frankfurt-Sachsenhausen**	**FS**	-	-	4	2 (2;8)	**Wismar**	**WI**	6	2 (1;7)	-	-
**Fuldatal**	**FT**	2	1 (1)	-	-						

### Haplotype network reconstruction of *Culex pipiens*

**Figure 2:**
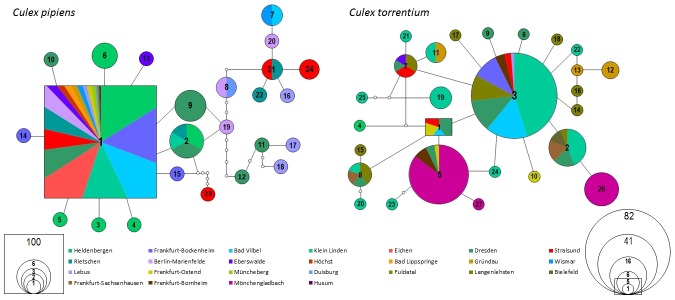
Haplotype networks of *Culex pipiens* and *Culex torrentium* for the cox1 gene segment calculated using statistical parsimony as implemented in TCS 1.21. The squares stand for the most probable ancestral haplotypes, the circle for all other haplotypes. The Numbers are equal to the haplotypes of each species. Each line represents a single mutation while small white dots symbolize hypothetical missing haplotypes. The size of the circles and the square is proportional to the number of the occurring haplotypes. The number of individuals can be derived from the scale which is given in the figure. Different colors represent the different geographical sampling localities. The colored area is proportional to the occurrence at the respective site.

The statistical parsimony network calculated with TCS using 399 cox1 sequences of *Cx. pipiens* resulted in one single network with 24 different haplotypes and no subnetworks. The highest outgroup probability within the network was calculated for H1 which was the most frequent (n = 361 individuals) and observed at all sampling sites (Figure 2). One part of the network had a rather star like structure. This was a result of 10 different haplotypes being directly derived from haplotype H1 and differing only by one mutation from the ancestral one (H2, 3, 4, 5, 6, 9, 10, 13, 14 and 15). Further connections within the network started from haplotypes H2 and H9. One haplotype (H19), which is only represented by one individual from Berlin-Marienfelde, seems to be the initial point for a more branched part of the network consisting of eleven different haplotypes. Nearly all individuals that carry one of these 11 haplotypes originate in the eastern parts of Germany, with the exception of haplotypes H7, H8 and H15. Haplotype seven is shared between Bad Vilbel and Berlin, haplotype eight is shared between Duisburg and Berlin, whereas H15 was exclusively found in Frankfurt-Bockenheim. Additionally, this part of the network is also characterized by several missing hypothetical intermediate haplotypes. In general the network structure is characterized by a dominant haplotype H1 and a star like structure. In the eastern parts of Germany there seems to be much more genetic variability and structure within *Cx. pipiens*.

### Haplotype network reconstruction of *Culex torrentium*

The statistical parsimony network calculated with TCS using 198 cox1 sequences of *Cx. torrentium* resulted in one single network and no subnetworks. Even with fewer individuals (198) the dataset was partitioned into 27 haplotypes which implies a higher genetic variability within *Cx. torrentium* compared to *Cx. pipiens*. The structure of the network was not dominated by one single haplotype. In general the pattern was also rather star like with rare haplotypes (n = 10) that are directly derived from the most frequent one (H3, Figure 2). The haplotype network of *Cx. torrentium* shows a higher connectivity than the network of *Cx. pipiens*; nearly every haplotype is more or less directly linked to the next sampled haplotype (they differ only by one mutation from one another) and only five hypothetical haplotypes are missing. Haplotype three was found 82 times and was present at eight different localities across Germany. Furthermore this haplotype is linked to the second most frequent haplotype (H5) via H24 which differed only by one mutation from both. In contrast to the haplotype network of *Cx. pipiens*, the most frequent haplotype was not the ancestral one. In *Cx. torrentium* the most probable ancestral haplotype (H1) is represented by five individuals and was found only at four different localities (Frankfurt-Ostend, Bad Vilbel, Dresden and Stralsund). It also differed only by one mutation from the two most frequent haplotypes (H3 and H5) and was directly linked to both. Besides the most frequent haplotype H3, additional haplotypes occurred at high frequencies (H5: 41 individuals, H2: 16 individuals and H26: 14 individuals). The second most frequent haplotype, H5, occurred at four different localities and was mainly found in Mönchengladbach. Furthermore this haplotype occurred in central Germany (Frankfurt) and eastern Germany (Dresden). Additionally some of the more frequent haplotypes were exclusively found at single localities like H26, which only occurred at Mönchengladbach. The haplotypes H3 and H5 were much more frequent at central and western localities (H3: 61 individuals and H5: 39 individuals) than in the eastern parts of Germany (H3: 13 individuals, H5: 2 individuals) Furthermore, haplotype H8 followed a similar geographical pattern to haplotypes H3 and H5 (distributed in central, eastern and northern Germany).

### Population structure

**Figure 3:**
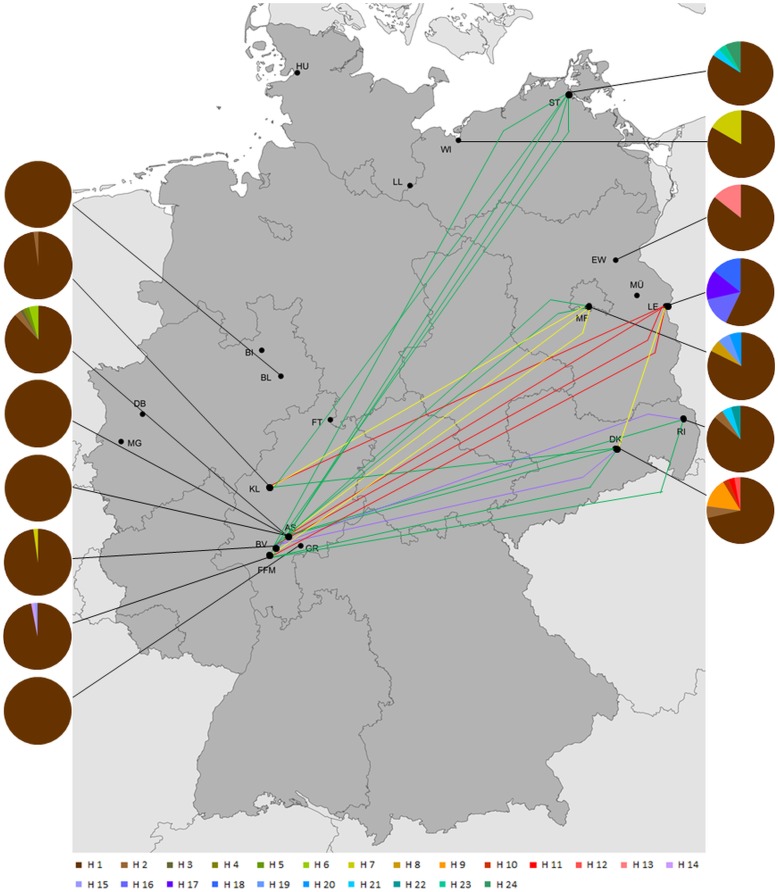
Sampling localities of *Culex pipiens* across Germany with significant different population pairwise F_ST_ values. Significant different pairwise F_ST_ values between populations are indicated using different line colors. Significant F_ST_ values were grouped into the four following categories: very great population differentiation (red lines), great population differentiation (yellow lines), moderate population differentiation (green lines) and low population differentiation (purple lines) [67]. Pictured are all sampling points listed in Table 4 with a summary of their haplotypes. Map was created with ArcMap 10.1.

**Figure 4:**
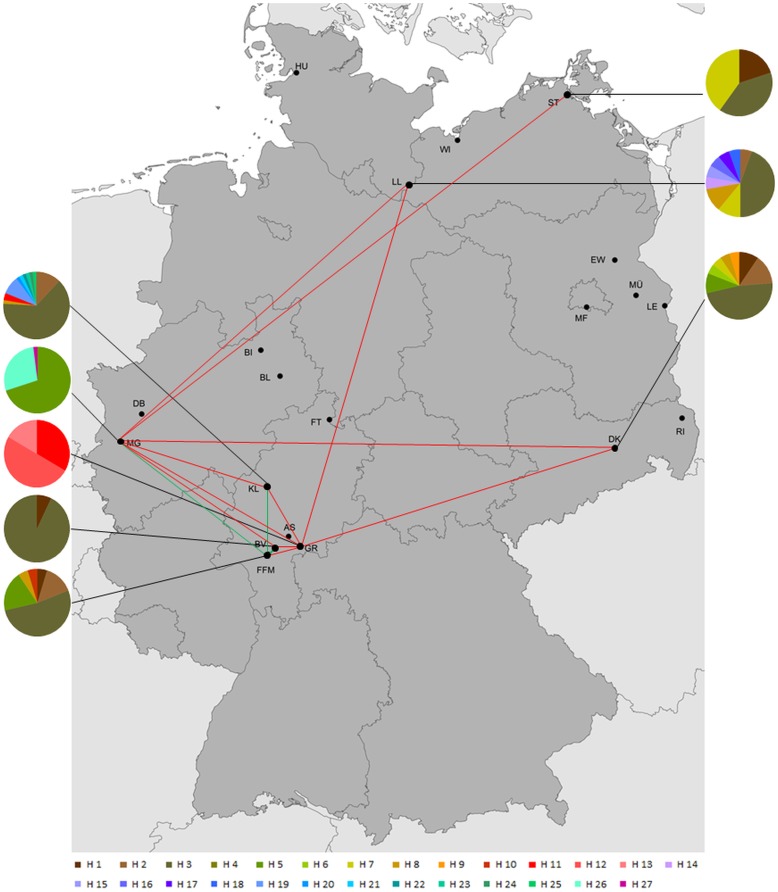
Sampling localities of *Culex torrentium* across Germany with significant different population pairwise F_ST_ values. Significant F_ST_ values were grouped into the four following categories: very great population differentiation (red lines), great population differentiation (yellow lines), moderate population differentiation (green lines) and low population differentiation (purple lines) [67]. Pictured are all sampling points listed in Table 5 with a summary of their haplotypes. There were no significant moderate or low F_ST_ values. Map was created with ArcMap 10.1.

Using the Tamura-3-parameter model, the analysis of genetic distances between both species resulted in a mean within group distance of 0.01% in *Cx. pipiens* and 0.03% in *Cx. torrentium*. The mean genetic distance between both species was 3%. The genetic differentiation based on population pairwise F_ST_ is shown in Tables 4 and 5. In addition the significant F_ST_ values are indicated in Figures 3 and 4, which also show the haplotype frequencies at the different sampling localities. In Figures 3 and 4 we also color coded the F_ST_ values into 4 categories [67]. These four categories were 1. very great population differentiation (red lines), 2. great population differentiation (yellow lines), 3. moderate population differentiation (green lines) and 4. low population differentiation (purple lines). In total 14 population comparisons of *Cx. torrentium* and *Cx. pipiens* showed significantly different pairwise F_ST_ values. Generally more populations were very greatly significantly differentiated in *Cx. torrentium* (n = 11, Figure 4) (according to the categories of Balloux and Lugon-Moulin [67]) than in *Cx. pipiens* (n = 5, Figure 3) but within *Cx. pipiens* a geographic pattern seemed to be more obvious. Central and eastern populations of *Cx. pipiens* were significantly differentiated and showed a moderate population differentiation, while in *Cx. torrentium* the degree of differentiation was higher and not only between central and eastern parts of Germany. Within *Cx. torrentium* the westernmost population (Mönchengladbach) was highly differentiated from all other populations except for Frankfurt-all with a moderate differentiation. Furthermore, significant genetic differentiation was detected in geographically much closer *Cx. torrentium* populations in Hesse (Figure 4 and Table 4).

**Table 4:** Population pairwise F_ST_ from *Culex pipiens* calculated with Arlequin 3.5.1.2.

Locality	AS 2	AS 1	BL	BV	GR	KL	FFM	AS 3	DK	EW	AS 3	MF	RI	ST	WI
**AS 2**	*														
**AS 1**	0.00000	*													
**BL**	0.00000	0.00000	*												
**BV**	−0.00323	−0.10987	−0.10987	*											
**GR**	0.00000	0.00000	0.00000	−0.10987	*										
**KL**	−0.00323	−0.10987	−0.10987	−0.00561	−0.10987	*									
**FFM**	−0.00751	−0.11041	−0.11041	0.00413	−0.11041	−0.00104	*								
**AS 3**	0.00376	−0.10057	−0.10057	0.00298	−0.10057	−0.00121	0.01082	*							
**DK**	**0.07564**	−0.06021	−0.06021	**0.04654**	−0.06021	**0.07288**	**0.09651**	**0.07461**	*						
**EW**	0.30534	−0.05528	−0.05528	−0.00563	−0.05528	0.16821	0.13919	0.01971	−0.00796	*					
**LE**	**0.48905**	0.06126	0.06126	**0.42131**	0.06126	**0.50400**	**0.56717**	**0.51701**	**0.18985**	0.11914	*				
**MF**	**0.16537**	−0.04209	−0.04209	**0.07339**	−0.04209	**0.15784**	**0.19920**	**0.14019**	0.00451	0.00866	0.12141	*			
**RI**	**0.09749**	−0.06598	−0.06598	**0.03721**	−0.06598	0.09313	**0.12442**	**0.08929**	0.01750	−0.01435	0.15050	−0.04295	*		
**ST**	**0.10595**	−0.05447	−0.05447	**0.06418**	−0.05447	**0.10750**	**0.13726**	**0.11439**	0.02827	−0.00910	0.10373	−0.03521	−0.03148	*	
**WI**	0.38291	−0.03448	−0.03448	0.15811	−0.03448	0.36754	0.41563	0.28445	0.02338	0.02173	−0.00011	−0.08272	−0.06711	−0.07826	*

Data based on the analyses of 389 sequences of the cox1 gene segment from 17 different sampling localities in Germany (FFM = Frankfurt/Main with KS, FB and FZ). Sample size are shown in Table 1, abbreviations of each locality are shown in Table 1 and 3. Significant different F_ST_ values are shown in bold. For calculation only populations with 5 or more individuals were used.

**Table 5:** Population pairwise F_ST_ from *Culex torrentium* calculated with Arlequin 3.5.1.2.

Locality	BV	FFM	GR	KL	MG	DK	ST	LL
**BV**	*							
**FFM**	**0.07117**	*						
**GR**	**0.59328**	**0.47067**	*					
**KL**	0.00030	**0.05859**	**0.48753**	*				
**MG**	**0.43031**	**0.25075**	**0.57234**	**0.42635**	*			
**DK**	0.02390	−0.03154	**0.45560**	0.01940	**0.30419**	*		
**ST**	0.27364	0.06676	0.32258	0.01877	**0.36069**	0.01428	*	
**LL**	0.01701	0.02913	0.36024	0.02495	**0.36577**	−0.00098	−0.03260	*

Data based on the analyses of 193 sequences of the cox1 gene segment from 10 different sampling localities in Germany (FFM = Frankfurt/Main with KS, FB and FZ). Sample size are shown in Table 1, abbreviations of each locality are shown in Table 1 and 3. Significant different F_ST_ values are shown in bold. For calculation only populations with 5 or more individuals were used.

The calculated AMOVA revealed that most of the genetic variability within *Cx. pipiens* was significantly explained by genotypic variation within populations (88.38%, Table 6). The grouping of the populations originating in western or eastern Germany additional explained a lower but significant part of the genetic variation (8.39%, Table 6). Only 3.32% of the genetic variation was explained by genotypic variation among populations within groups (see Table 6). The AMOVA for *Cx. torrentium* also revealed that the biggest part of the genetic variation was significantly explained by genotypic variation within populations (66.54%, Table 6), but in addition a higher degree of the genetic variation was explained by the variation among groups (32.42%, Table 6). The higher degree of differentiation within *Cx. torrentium* was also supported by the overall index of population differentiation (F_ST_) derived from the AMOVA calculations where the F_ST_ value of *Cx. torrentium* (0.33) is much higher than the F_ST_ value within *Cx. pipiens* (0.11).

**Table 6:** Results of the Analysis of molecular variance (AMOVA) and level of genetic differentiation of *Culex pipiens* and *Culex torrentium* measured by F_CT_, F_SC_, and F_ST_.

*Culex pipiens*							*Culex torrentium*						
Source of variation	d.f.	Sum of squares	Variance components		Percentageof variation	p-value	Source of variation	d.f.	Sum of squares	Variance components		Percentage of variation	p-value
**Among groups**	1	5.492	0.02924	Va	8.39	p<0.05	**Among groups**	3	42.486	0.31775	Va	32.42	p<0.05
**Among populations within groups**	13	7.556	0.01126	Vb	3.32	n.s.	**Among populations within groups**	4	3.383	0.01019	Vb	1.04	n.s.
**within populations**	374	115.204	0.30803	Vc	88.38	p<0.05	**within populations**	185	120.650	0.65216	Vc	66.54	p<0.05
**Total**	388	128.252	0.34853				**Total**	192	166.519	0.98010			

All Data were calculated in Arlequin 3.5.1.2 [59] based on the distance method of Tamura and Nei [60].

As there were indications of genetic differentiation between populations from central and eastern Germany within *Cx. pipiens* and also some indications for strong genetic differentiation between populations from the western parts of Germany and central and east-German populations of *Cx. torrentium* (see above), we tested for isolation by distance using distance based redundancy analysis (dbRDA). However, only a very low proportion of the genetic variation could be significantly explained by geographical distance; the spatial coordinates only explained 2% of the genetic variability in *Cx. pipiens* and 5% of the genetic variation within *Cx. torrentium*. The ratio of non-synonymous to synonymous polymorphisms within *Cx. pipiens*/*Cx. torrentium* and within the outgroup was not significantly different from the ratio of non-synonymous to synonymous polymorphisms fixed between these groups (Fisher's exact test P = 0.337/0.380). Thus, the McDonald-Kreitman test is consistent with neutral evolution of the cox1 gene and there were no indications that there is selection acting on the studied mitochondrial gene fragment. To reveal signs of population expansion, signs of bottleneck within the population or sudden contractions, we used Tajima's D for which the level of significance was assessed using the permutation test implemented in Arlequin. The overall Tajima's D for *Cx. pipiens* was negative (−0.75 +/− 0.73) but non-significant (p>0.05). For *Cx. torrentium* the overall mean Tajima's D was only slightly negative (−0.13+/−1.03) and also non-significant (p>0.05). All calculations for *Cx. torrentium* populations resulted in non-significant Tajima's D which was either strongly positive or negative. In contrast several populations of *Cx. pipiens* showed a strongly negative Tajima's D (Heldenbergen:−1.77; Bad Vilbel: −2.18 and Frankfurt-Bockenheim: −1.45; all p-values>0.05). These highly negative Tajima's D might indicate a recent demographic or range expansions within species [64], [65] either after a bottleneck (e.g. [63]) or a selective sweep [57].

## Discussion

Information about the genetic variability of *Cx. torrentium* and *Cx. pipiens* is of particular interest as both species are ornithophilic and potential enzootic vectors for certain arboviruses [68]. In total 597 individuals of both species from 25 localities in Germany were analysed to reveal distribution patterns of these two sibling species in Germany. *Culex pipiens* could be detected at 21 out of 25 sampling sites whereas *Cx. torrentium* was only found at 15 out of 25 sampling localities. One explanation for the dominance of *Cx. pipiens* at several localities could be the different developmental rates of both species which can lead to approximately one generation less per year in *Cx. torrentium* [3], [69]. Another factor that might have impacted the results is temperature. The development of *Cx. pipiens* was described to take place between temperature ranges of 8–30°C [3], but in Sweden the species could only be found in areas with a mean temperature of 11.9°C from May to August 1950–2000. In the latter study *Cx. torrentium* was found in areas with a mean temperature of 10.5°C for May to August 1950–2000 [22]. Taking the influence of temperature into account, the species composition at different sites can vary within and between the years, depending on the actual but also the mean temperature. In our survey *Cx. torrentium* was often found in artificial water bodies. Although this has been described previously [12], it contradicts the common conception that *Cx. torrentium* utilizes more natural larval habitats than *Cx. pipiens* [45]. Furthermore, larvae of *Cx. pipiens* as well as of *Cx. torrentium* were found, often sympatrically, in various kinds of habitats. Therefore, it seems unlikely that habitat constitutes an important factor for species composition at the localities, as both species were equally observed in different habitats.

The genetic analyses of populations of *Cx. torrentium* and *Cx. pipiens* provide insights into the genetic structure of these potential disease vectors in Germany. While *Cx. pipiens* with its bioforms *Cx. pipiens pipiens* and *Cx. pipiens* form *molestus* belongs to the worldwide distributed *Cx. pipiens* complex, *Cx. torrentium* is characterized as a sibling species [28]. All of these species are known to be important vectors for various arboviruses and exhibit various behavioral differences [14] (e.g. the host preference), which are important with regard to their particular vector-competence. Due to the limited knowledge about the genetic structure of these species, it is unknown whether these behavioral modifications result from genetic variation or genetic polymorphism. In this context hybrids between ornithophilic and anthropophilic species are of particular interest, as they could serve as important bridge vectors for diseases like West Nile fever. In Germany such a hybrid was described just recently for the first time [28].

To avoid generalizations about species' vector biology, more knowledge about their spatial genetic composition is crucial as different populations could vary with regard to their vectorial abilities. Different species or even populations may also hybridize with each other, which could result in varying biting behavior concerning the host preference. Therefore, population genetic studies are essential for evaluation of the respective roles members of the *Cx. pipiens* complex and *Cx. torrentium* play in enzootic and/or epidemic transmission of arboviruses in Europe. A clear identification of *Cx. pipiens* form *pipiens*, *Cx. pipiens* form *molestus* and *Cx. torrentium* is essential but due to the difficulties in morphological identification, molecular methods are necessary to distinguish these forms. Today, commonly used techniques are the amplification of the cox1 gene fragment followed by restriction enzyme digestion of the amplicons [12], [70] or single-nucleotide polymorphism (SNP) analysis [71], as well as the second intron of the acetylcholinesterase- (ACE-2) or ITS-sequences of rDNA-based PCR assays and different allozyme markers [15], [72], [73]. *Culex pipiens* and *Cx. torrentium* are treated in many studies just as bundles of “*Cx. pipiens/torrentium*” and have not been separated, leading to the point that not many population structure analyses of these species have been performed in the past for Europe [16]. In contrast to a study on species in the Frankfurt/Rhine-Main Metropolitan Region, where *Cx. torrentium* showed a higher genetic diversity than *Cx. pipiens* [44], the recent study provides a similar high amount of variation for both species with 27 and 24 haplotypes, respectively. It is striking to note, that in total more haplotypes could be identified from *Cx. torrentium*, but some haplotypes of *Cx. pipiens* provide more substitutions within the analysed fragment. The much lower genetic differentiation of *Cx. pipiens* populations is most likely due to the observed dominant and widespread haplotype H1 which occurred at every sampling locality. The structure of the network with one dominant haplotype and many rare haplotypes (see Figure 2) in combination with some populations showing a significant highly negative Tajima's D, might suggest a population-wide demographic [74] or a recent range expansion [64], [65]. The population structure of *Cx. pipiens* might also be explained by other reasons such as a recent bottleneck (e.g. [63]) or a selective sweep [57]. Both of these events could be the result of genetic drift with in certain populations and could lead, as seen in the case of *Cx. pipiens*, to a reduced overall genetic variability. The highest genetic variability was explained by variation within populations (see Table 6) whereas isolation by distance was not the main reason for the genetic variability (as only 2% of the genetic variability was explained by spatial variables). Thus, the populations themselves are variable but there were no clear differences between populations according to the haplotype distribution across Germany (Figure 3).

It is also obvious that there is a moderate differentiation between western and eastern populations of *Cx. pipiens* in Germany (Figure 3 and Table 3). The reason for this might be that *Cx. pipiens* occurs in two bioforms (*Cx. pipiens* form *pipiens* and *Cx. pipiens* form *molestus*) and that these bioforms differ in their relative abundance in different parts of Germany. Former studies [28] could not detect *Cx. pipiens* form *molestus* in the eastern part of Germany; nevertheless the appearance of this form cannot be excluded and could be an explanation for the observed higher haplotype diversity in eastern populations.

One main difference between the population structure of *Cx. pipiens* and *Cx. torrentium* is the much higher genetic differentiation between populations of *Cx. torrentium*; the overall F_ST_ value was 0.33 for *Cx. torrentium* compared to a three times lower F_ST_ value of *Cx. pipiens* (F_ST_ = 0.11). Furthermore the absence of one single dominant haplotype, the higher haplotype diversity, the stronger genetic differentiation between populations (see Figure 4) and the relative amount of genetic variability which is explained by the AMOVA among groups (see Table 6) reflects the different population structure. The high percentage of genetic variability which is explained by the defined groups in the AMOVA (>30%) is mainly due to the strong differentiation of the most western population (Mönchengladbach) and one central population (Gründau-Rothenbergen) in comparison to the remaining populations. The population Mönchengladbach is represented by many individuals (n = 50) but there was only one haplotype that was shared between Mönchengladbach and two other sampling sites, which than led to a strong differentiation. As Gründau-Rothenbergen (GR) is only represented with five individuals the strong differentiation based on the categories by Balloux and Lugon-Moulin [67] and based on the AMOVA should be interpreted with caution. Additional sampling from this locality could lead to a detection of more widespread haplotypes and thus could lower the degree of differentiation. The observed population structure of *Cx. torrentium* might indicate lower dispersal capacities than in *Cx. pipiens* which leads to a reduced gene flow between populations and thus might explain the stronger differentiation. On the other hand, a more recent demographic or range expansion within *Cx. pipiens* could also explain the observed differences between both species. In addition, a higher amount of genetic variation is explained by the dbRDA analysis (2% within *Cx. pipiens*; 5% within *Cx. torrentium*). As the dbRDA is based on pairwise genetic distances between individuals and geographic coordinates, all individuals from every sampling site were included in this analysis.

Previous studies have shown that the mean dispersal range of *Culex* spp. is about 0.2–2.6 km, depending on various factors [75], [76]. The dispersal depends on the habitat, being lower in residential than in rural areas [77]. Nevertheless, other authors estimate a mean dispersal range for *Cx. erraticus* of 0.967 km which is close to the mean range by mark-release-recapture experiments (0.73 km) [78]. This range characterizes *Cx. erraticus* as a stronger flyer than most other mosquito species that disperse only a few hundred meters. In our study, Frankfurt city center with many skyscrapers and very patchy distributed larval habitats seems to serve as a barrier between the localities FFM-Ostend (FZ), FFM-Bornheim (FB) and FFM-Bockenheim (KS). Between these localities and for FFM-Sachsenhausen (FS) the Main River serves as an additional geographic barrier which can limit both geographic as well as genetic expansion. Furthermore, the habitat of FFM-Sachsenhausen differs from the others sampling stations, as it is located in a rural, forested area. As stated by LaPointe [79] in his study on *Cx. quinquefasciatus* undertaken in a forest, dispersal is the result of appetential flights, searching for hosts or oviposition sites. Here, both species were found in municipal and in rural areas, respectively. Within the urban localities the highest numbers of specimens were found at places with a slight suburban character like gardens or patios. This indicates that *Cx. pipiens* as well as *Cx. torrentium* both have adapted to a life in human neighborhoods. Suitable conditions for reproduction are met in these environments; artificial containers serve as egg deposits for both species (own observation) and hosts for blood meals are frequent, i.e. humans and synanthropic birds e.g. sparrows, pigeons and blackbirds. Therefore long appentential flights are not necessary in these areas, which might explain the differentiation between the *Cx. torrentium* populations within Frankfurt. Furthermore, the population structure of *Cx. pipiens* and the lower genetic differentiation than in *Cx. torrentium* might be due to the infection with different strains of *Wolbachia* symbionts. Not all populations are compatible with each other. Crossings of males from a southern German population with females from the North result in fertile descendants whereas reciprocal crosses, on the other hand, result in fertile clutches but no hatchings, indicating a reproduction barrier [80] as the result of cytoplasmic incompatibilities (CI) due to infection by *Wolbachia* symbionts. Additionally, several factors such as the *Wolbachia* strain, amount of infection, host species, temperature and rearing density influence the CI intensity [81]–[84]. In populations with infected and uninfected mosquitoes, *Wolbachia*-free females are at a reproductive disadvantage when they copulate with infected males; the spread and fixation of *Wolbachia* infections is facilitated [85] and leads to an increase in the frequency of *Wolbachia* with each generation. As such an infection can sweep through the population leading to reduced mitochondrial diversity [86]. This can explain why this species shows one dominant haplotype throughout all populations. Although 23 other haplotypes have been detected, most of these were very scarce and displayed only by one or two specimens. It would be interesting, however, to investigate, whether specimens that show the highest percentage of various haplotypes (localities Dresden and Lebus and to a lesser degree Heldenbergen), exhibit different *Wolbachia* strains resulting in CI or a higher degree of uninfected specimens. Two problems which could lead to misinterpretation of the results are pseudogenes and maternally inherited symbionts, like *Wolbachia*. While the situation for *Cx. pipiens* has been discussed already, the situation for *Cx. torrentium* is different, as pseudogenes can be excluded because of the previous testing of the dataset. No *Wolbachia* infections have been found so far in *Cx. torrentium* [87]. If *Wolbachia* infections occur in German populations of *Cx. torrentium*, it could mean that there are no reproductive barriers resulting from CI and no mitochondrial sweep through populations, which explains the heterogeneity of most of the analysed populations.

## Conclusion

As shown here, *Cx. pipiens* and *Cx. torrentium* are synanthropic and share similar ecological habitats, which in some cases contradict the common conception. One example of this is the larval habitat. *Culex torrentium* was thought to utilize more natural breeding sites, whereas in this study larvae of both species were often found sympatric in artificial water bodies. However, different factors underlie the mitochondrial differentiation in both species. Some studies argued that feral and synanthropic forms seem to be sufficient for gene flow to diminish drift in *Cx. pipiens* [88]. The relatively low differentiation between most populations observed here might be explained by a sweep, resulting from insecticide resistance after eradication programs. Furthermore, a low differentiation can be induced by endosymbionts such as *Wolbachia* or insecticide-resistance due to previous eradication events. Another aspect leading to decreased genetic differentiation may be the dispersal between nearly located populations or mixing by human introduction. In the case of *Cx. torrentium* these mechanisms seem to act in the opposite direction: dispersal as well as human introduction influences the mitochondrial diversity of populations. In previous works, *Wolbachia* could not be detected in *Cx. torrentium* [87]. Therefore, a sweep as in *Cx. pipiens* has not occurred and the mitochondrial diversity of populations could remain constant or even increase.

Nevertheless, the species show a big natural diversification. The very strong differentiation between populations indicates a splitting within *Cx. torrentium* with a higher haplotype diversity as well as the absence of one dominant haplotype which was only found for *Cx. pipiens*. Different species can vary in their insecticide resistance, ecological habits and vector competence. However, whether cryptic species are involved, or *Cx. torrentium* might be a variable species should be the focus of future studies. Hybrids between the two bioforms *pipiens* and *molestus* and between *molestus* and *torrentium* are of particular interest, as these represent potentially important bridge vectors for different zoonotic arboviruses possibly having a major impact in the risk assessments for arboviruses in Germany. It has to be noted that due to possible insecticide-resistance or *Wolbachia*-induced sweeps in populations, mitochondrial markers are not, or just in a limited way, useful for analyses of such infected populations. On the other hand, mitochondrial markers are generally producing reliable results for species without mitochondrial population-sweeps. Another explanation for the low haplotype diversity can be the result of insecticide-induced sweeps after eradication programs, when only a small percentage of the original population remains. Apart from the aforementioned remaining questions, future studies should focus on dispersal for both species in different areas as different dispersal capabilities can result in varying differentiation and possibly even in distinct vector competences for different populations.

## Supporting Information

Table S1 **Names and contact details of all private persons can and will be given on request.**
(DOCX)
