# Supplementary material for: Population Structure and Distribution Patterns of the Sibling Mosquito Species Culex pipiens and Culex torrentium (Diptera: Culicidae) Reveal Different Evolutionary Paths
Source: PLoS One. 2014 Jul 21;9(7):e102158. doi: 10.1371/journal.pone.0102158 (PMC4105623; doi:10.1371/journal.pone.0102158)
Supplement: Table S1 — Names and contact details of all private persons can and will be given on request. (DOCX) [file pone.0102158.s001.docx]

Table S1 Names and contact details of all private persons can and will be given on request.

| **Locality** |  |
| --- | --- |
| **Bad Lippspringe** | private person; no specific permissions required |
| **Bad Vilbel** | Jugend forscht project (Marc Grahmann) |
| **Berlin-Marienfelde** | private person; no specific permissions required |
| **Bielefeld** | private person; no specific permissions required |
| **Dresden** | Dr. Andreas Weck-Heimann (Senckenberg Dresden) |
| **Duisburg** | private person; no specific permissions required |
| **Eberswalde** | private person; no specific permissions required |
| **Eichen** | Public ground; no specific permissions required |
| **Frankfurt-Bockenheim** | area Senckenberg institute; no specific permissions required |
| **Frankfurt-Bornheim** | Cemetery Bornheim (Donato Marrone) |
| **Frankfurt-Ostend** | Zoo Frankfurt (Prof Dr. Manfred Niekisch) |
| **Frankfurt-Sachsenhausen** | Frankfurt Stadtwald; no specific permissions required |
| **Fuldatal** | private person; no specific permissions required |
| **Gründau-Rothenbergen** | private person; no specific permissions required |
| **Heldenbergen** | Public ground; no specific permissions required |
| **Höchst a.d. Nidda** | Public ground; no specific permissions required |
| **Husum** | private person; no specific permissions required |
| **Klein Linden** | private person; no specific permissions required |
| **Langenlehsten** | BNITM |
| **Lebus** | Arne Köhler (Senckenberg Müncheberg) ; no specific permissions required |
| **Mönchengladbach** | private person; no specific permissions required |
| **Müncheberg** | Landrat Märkisch-Oderland, permission AZ: 32.45/36-12-0009 |
| **Rietschen** | private person; no specific permissions required |
| **Stralsund** | private person; no specific permissions required |
| **Wismar** | private person; no specific permissions required |
|  |  |

No endangered or protected species are involved in this study.
